# Inverse Correlation Between Coffee Consumption and Prevalence of Metabolic Syndrome: Baseline Survey of the Japan Multi-Institutional Collaborative Cohort (J-MICC) Study in Tokushima, Japan

**DOI:** 10.2188/jea.JE20120053

**Published:** 2013-01-05

**Authors:** Hidenobu Takami, Mariko Nakamoto, Hirokazu Uemura, Sakurako Katsuura, Miwa Yamaguchi, Mineyoshi Hiyoshi, Fusakazu Sawachika, Tomoya Juta, Kokichi Arisawa

**Affiliations:** Department of Preventive Medicine, Institute of Health Biosciences, The University of Tokushima Graduate School, Tokushima, Japan; 徳島大学大学院へルスバイオサイエンス研究部予防医学分野

**Keywords:** metabolic syndrome, coffee, serum triglycerides, plasma glucose, green tea

## Abstract

**Background:**

It is unclear whether consumption of coffee and green tea is associated with metabolic syndrome.

**Methods:**

This cross-sectional study enrolled 554 adults who had participated in the baseline survey of the Japan Multi-Institutional Collaborative Cohort (J-MICC) Study in Tokushima Prefecture, Japan. Consumption of coffee and green tea was assessed using a questionnaire. Metabolic syndrome was diagnosed using the criteria of the National Cholesterol Education Program Adult Treatment Panel III (NCEP ATP III) and the Japan Society for the Study of Obesity (JASSO). Logistic regression analysis was used to examine the association between consumption of coffee and green tea and prevalence of metabolic syndrome and its components.

**Results:**

After adjustment for sex, age, and other potential confounders, greater coffee consumption was associated with a significantly lower prevalence of metabolic syndrome, as defined by NCEP ATP III criteria (*P* for trend = 0.03). Participants who drank more coffee had a lower odds ratio (OR) for high serum triglycerides (*P* for trend = 0.02), but not for increased waist circumference or high blood pressure. Using JASSO criteria, moderate coffee consumption (1.5 to <3 cups/day) was associated with a significantly lower OR for high plasma glucose (OR = 0.51, 95% CI 0.28-0.93). Green tea consumption was not associated with the prevalence of metabolic syndrome or any of its components.

**Conclusions:**

Coffee consumption was inversely correlated with metabolic syndrome diagnosed using NCEP ATP III criteria, mainly because it was associated with lower serum triglyceride levels. This association highlights the need for further prospective studies of the causality of these relationships.

## Abstract (ja)

**【背景】:**

コーヒーおよび緑茶の摂取量とメタボリックシンドローム (MetS)との間に関連があるかどうかは明らかになっていない。

**【方法】:**

この断面調査では、日本多施設共同コーホート研究 (J-MICC Study)徳島地区ベースライン調査に参加した554名を対象とした。コーヒーと緑茶の摂取量は、質問票で測定した。MetSの診断には、National Cholesterol Education Program Adult Treatment Panel Ⅲ (NCEP ATPⅢ)および日本肥満学会 (JASSO)の基準を用いた。コーヒーおよび緑茶の摂取量とMetSおよびその構成因子の有病率との間の関連を調べるためにロジスティック回帰分析を用いた。

**【結果】:**

性、年齢、その他の交絡因子を調整した後、コーヒー摂取量とNCEP ATPⅢのMetS基準により診断したMetSの有病率との間で有意な負の関連を認めた (P for trend= 0.03)。またコーヒーの高摂取群で中性脂肪高値のオッズ比 (OR)の有意な低下を認めたが (P for trend= 0.02)、腹囲の増加や血圧の上昇との関連は認めなかった。JASSOの基準を用いた場合は、コーヒーの中摂取群 (1.5杯/日以上3杯/日未満)で高血糖のオッズ比の有意な低下を認めた (OR= 0.51、95％信頼区間: 0.28-0.93)。緑茶の摂取量とMetSおよびその構成因子との間には有意な関連を認めなかった。

**【結論】:**

コーヒー摂取が、主として血清中性脂肪濃度との負の相関により、NCEP ATPⅢ基準によるMetSの有病率の低下と関連していることが示唆された。今後、因果関係の検証のため、前向き研究などのさらなる研究が必要である。

## INTRODUCTION

Metabolic syndrome (MetS) is characterized by the clustering of abdominal obesity, impaired glucose tolerance, elevated triglyceride levels, reduced high-density lipoprotein (HDL) cholesterol levels, and hypertension, often accompanied by a proinflammatory state.^1^ Persons with MetS are at increased risk of cardiovascular diseases and type 2 diabetes, not only in Western^2^ but also in Asian populations.^3^^,^^4^ Although less frequent than in Western countries, obesity and MetS are also major health problems in Japan. According to the 2008 National Nutritional and Health Survey, the prevalence of MetS in Japan was estimated at 25.3% for men and 10.6% for women.^5^

Worldwide, MetS has been diagnosed using several different sets of criteria, such as those of the National Cholesterol Education Program Adult Treatment Panel III (NCEP ATP III),^1^ the International Diabetes Federation,^6^ and the Japan Society for the Study of Obesity (JASSO).^3^^,^^7^ These definitions differ somewhat in terms of the prerequisite components and the cut-off levels used for each component.

Lifestyle factors reported to be associated with an increased risk of MetS include a diet with a high intake of refined grains, meat, and fried foods^8^; low diet quality, ie, high fat and low dietary fiber and vitamins^9^; and smoking,^10^ whereas a diet with a high intake of vegetables and fruits,^11^ and high levels of leisure-time physical activity,^10^^,^^12^ are believed to be protective.

Recently, habitual consumption of coffee^13^^,^^14^ was reported to be a protective factor against development of type 2 diabetes. However, the association between coffee consumption and MetS remains unclear. Two cross-sectional studies in Japan showed inverse correlations of coffee consumption with MetS and some of its components,^15^^,^^16^ while prospective studies performed in the United States^8^ and Europe^10^^,^^17^ showed no association.

Previous epidemiologic studies performed mainly in Asian countries showed that high intake of tea, green tea, or green tea extracts was associated with decreases in body weight and waist circumference,^18^ serum triglycerides,^19^ blood pressure,^20^ and diabetes incidence.^21^ However, an association between green tea consumption and MetS has not been proven.

In the present report, we examined the correlation between consumption of coffee and green tea and the prevalence of MetS and its components, as defined using the criteria of NCEP ATP III and JASSO.

## METHODS

### Study participants

The population of the present study consisted of 577 men and women aged 35 to 70 years who had attended the Tokushima Prefectural General Health Check-up Center from January 23, 2008 to November 24, 2011 and had participated in the baseline survey of the Japan Multi-Institutional Collaborative Cohort (J-MICC) Study. Of 3911 subjects who had received a medical examination during the study period and were asked to participate in the J-MICC Study, 577 (14.8%) agreed. We excluded people with a previous history of stroke (*n* = 5) or ischemic heart disease (*n* = 8) and those whose health check-up data were not available (*n* = 10), which left 554 participants (409 men and 145 women) for the present analysis. The details of the J-MICC Study have been described in a previous report.^22^ Briefly, the purpose of the J-MICC Study was to examine prospectively the associations of lifestyle and genetic factors and their interactions with the risk of chronic diseases. After thoroughly explaining the outline and purpose of the study, written informed consent was obtained from each participant. The study protocol was reviewed and approved by the ethics committees of Nagoya University School of Medicine (the affiliation of the former principal investigator, Dr. N. Hamajima), Aichi Cancer Center (the affiliation of the present principal investigator, Dr. H. Tanaka), and Tokushima University Hospital.

### Questionnaire

Each study participant was requested to answer a self-administered questionnaire that included items on current and previous diseases, physical activity, frequency of intake of foods and beverages, and smoking and drinking habits. Regarding dietary habits, participants were asked how often they had consumed 53 items of foods and beverages during the previous year. The questionnaire included 2 items on coffee consumption, namely, did the participants drink (1) filtered or instant coffee, and did they drink (2) canned, bottled, or packed coffee. The frequency of coffee intake was divided into 7 categories: rarely, 2 cups or less/week, 3–4 cups/week, 5–6 cups/week, 1–2 cups/day, 3–4 cups/day, and 5 cups or more/day. The total frequency of coffee intake was calculated as the sum of filtered or instant coffee and canned, bottled, or packed coffee. The questionnaire also included 1 item on Japanese green tea consumption (green tea, coarse tea, premium green tea, etc.). The level of intake was classified into 5 categories: rarely, 1–3 cups/day, 4–6 cups/day, 7–9 cups/day, and 10 cups or more/day. Total energy intake and percent energy from fat were calculated using a program developed at the Department of Public Health, Nagoya City University School of Medicine.^23^ Physical activity during leisure time was estimated by multiplying frequency and duration of light exercise (walking, etc.; 3.4 metabolic equivalents [METS]), moderate exercise (jogging, swimming, etc.; 7.0 METS), and heavy exercise (martial arts, marathon, etc.; 10.0 METS). These values were then summed and expressed as MET-hours/week.

The validity of the food frequency questionnaire was examined by comparing the questionnaire findings with data from a 3-day diet record that were collected after an interval of 1 month or shorter, in 29 participants. The Spearman rank correlation coefficient between the frequency of intake obtained from the questionnaire and that obtained from the diet record was 0.51 for coffee and 0.55 for green tea.

### Diagnosis of MetS

Data on anthropometric measurements (height, weight, and waist circumference), blood pressure, fasting plasma glucose, and serum triglycerides and high-density lipoprotein (HDL) cholesterol were obtained at the time of routine health check-ups. Participants were requested not to eat after 8:00 PM the preceding evening and received their medical check-up during the period from 8:00 AM to 11:30 AM. For 26 participants with missing data on waist circumference, self-reported data from the questionnaire were used instead. A diagnosis of MetS was made by using NCEP ATP III^1^ and JASSO^3^^,^^7^ criteria. When using the NCEP ATP III criteria, participants were diagnosed as having MetS when they satisfied at least 3 of the following 5 criteria: waist circumference of at least 90 cm in men or at least 80 cm in women; serum triglycerides of at least 150 mg/dl; HDL cholesterol less than 40 mg/dl in men or less than 50 mg/dl in women; systolic blood pressure of at least 130 mm Hg and/or a diastolic blood pressure of at least 85 mm Hg, or hypertension treatment; and fasting plasma glucose of at least 100 mg/dl or diabetes treatment. When using the JASSO criteria, participants were diagnosed as having MetS when they had a waist circumference of at least 85 cm in men or at least 90 cm in women and at least 2 of the following 3 criteria: serum triglycerides of at least 150 mg/dl and/or HDL cholesterol less than 40 mg/dl; systolic blood pressure of at least 130 mm Hg and/or diastolic blood pressure of at least 85 mm Hg, or hypertension treatment; and fasting plasma glucose of at least 110 mg/dl or diabetes treatment.

### Statistical analysis

The associations of coffee and green tea consumption with the prevalence of MetS and its components were examined using multiple logistic regression analysis, after adjustment for sex, age (continuous), total energy intake (quartiles), leisure-time physical activity (quartiles), and smoking (current, no, ex-smoker) and drinking habits (current drinker, other). In the analysis of green tea consumption, the frequency of coffee consumption was considered as a potential confounder and additionally adjusted for. The amount of coffee consumption was re-classified into 3 groups (<1.5 cups/day, 1.5 to <3 cups/day, ≥3 cups/day), and green tea consumption was re-classified into 3 categories (<1 cup/day, 1 to ≤3 cups/day, ≥4 cups/day), so that the numbers of participants in the categories were as close to equal as possible. The category with the lowest intake was used as the reference in both analyses. Odds ratios (ORs) and profile likelihood 95% CIs are presented. The dose-response relationships of coffee and green tea consumption with MetS were assessed by using ordinal categorical variables of 1, 2, and 3 for each category of consumption. Likelihood-ratio tests were performed in this analysis. A 2-tailed *P* value of less than 0.05 was considered significant. All statistical analyses were done using the SAS software package (version 8.2).^24^

## RESULTS

Table 1 shows the descriptive data for age, anthropometric measurements, blood pressure, blood biochemical tests, total energy intake, physical activity, frequency of coffee and green tea consumption, and smoking and drinking habits, according to sex. Mean age was 52.2 years for men and 52.4 years for women. Median frequency of coffee consumption was 11.5 cups/week for men and 10.6 cups/week for women. Median green tea consumption was 2 cups/day for men and women.

**Table 1.** Characteristics of the study participants

	Men (No. = 409)	Women (No. = 145)
Age (years)^a^	52.2 (9.4)	52.4 (9.1)
Height (cm)^a^	169.3 (5.9)	156.6 (6.1)
Weight (kg)^a^	69.5 (11.1)	55.5 (7.4)
Body mass index (kg/m^2^)^a^	24.2 (3.3)	22.7 (3.2)
Waist circumference (cm)^a^	86.0 (9.0)	80.3 (9.3)
Systolic blood pressure (mm Hg)^a^	120.1 (14.6)	115.3 (15.2)
Diastolic blood pressure (mm Hg)^a^	73.7 (10.6)	68.5 (10.5)
Triglycerides (mg/dl)^b^	103 (73, 149)	79 (59, 109)
High-density lipoprotein cholesterol (mg/dl)^a^	55.5 (16.6)	65.9 (14.4)
Fasting plasma glucose (mg/dl)^a^	102.7 (23.2)	97.9 (24.6)
Total energy intake (kcal/day)^a^	1902 (332)	1539 (220)
Physical activity (MET-hours/week)^b^	7.7 (1.3, 21.9)	5.1 (0.4, 15.3)
Coffee consumption (cups/week)^b^	11.5 (5.6, 24.6)	10.6 (3.6, 11.5)
Green tea consumption (cups/day)^b^	2 (2, 2)	2 (2, 5)

Smoking habit (No., %)		
Current	109 (26.7)	12 (8.3)
Past	169 (41.3)	15 (10.3)
No	131 (32.0)	118 (81.4)
Drinking habit (No., %)		
Current	306 (74.8)	59 (40.7)
Past	7 (1.7)	2 (1.4)
No	96 (23.5)	84 (57.9)

^a^Mean (SD).

^b^Median (25%, 75%).

Participants with a higher level of coffee consumption were younger and more likely to be male (data not shown). After adjustment for age and sex, participants who drank more coffee also had lower systolic and diastolic blood pressures. In addition, the proportion of current smokers was higher among those who drank more coffee. Frequency of coffee consumption was weakly inversely correlated with green tea consumption.

Table 2 shows sex- and age-adjusted and multivariate-adjusted associations of coffee consumption with the prevalence of MetS and its components, as defined using NCEP ATP III criteria. The overall prevalence of MetS was 114/554 (20.6%). After adjustment for sex, age, total energy intake, physical activity, and smoking and drinking habits, participants who drank more coffee (1.5 to <3 cups/day or ≥3 cups/day) had significantly lower ORs for MetS than did the reference group (<1.5 cups/day; *P* = 0.03 and 0.03, respectively). Greater coffee consumption (≥3 cups/day) was also associated with a significantly lower prevalence of high serum triglycerides (≥150 mg/dl; OR = 0.53, 95% CI 0.31–0.90), but not with increased waist circumference, high blood pressure, or high fasting plasma glucose. However, when blood pressure was treated as a continuous variable and treatment for hypertension was additionally adjusted for in multiple regression analysis, a coffee intake of at least 3 cups/day was associated with a change of −4.4 (95% CI −7.2 to −1.5) mm Hg in systolic blood pressure (*P* = 0.003) and a change of −2.3 (95% CI −4.5 to −0.1) mm Hg in diastolic blood pressure (*P* = 0.03). There was a dose-response relationship with coffee consumption for the prevalence of MetS (*P* = 0.03) and increased serum triglycerides (*P* = 0.02).

**Table 2.** Associations of coffee consumption with the prevalence of metabolic syndrome and its components according to NCEP ATP III criteria (No. = 554)

	Coffee consumption (cups/day)			*P* for trend

	<1.5	≥1.5 and <3	≥3	
Metabolic syndrome				
No. of cases/subjects	47/168	34/193	33/193	
Adjusted^a^ OR (95% CI)	1.00	0.60 (0.36–0.99)	0.61 (0.36–1.03)	0.06
Adjusted^b^ OR (95% CI)	1.00	0.56 (0.33–0.95)	0.55 (0.31–0.94)	0.03

Waist circumference ≥90 cm in men and ≥80 cm in women				
No. of cases/subjects	62/168	73/193	72/193	
Adjusted^a^ OR (95% CI)	1.00	1.02 (0.66–1.60)	1.28 (0.82–2.03)	0.27
Adjusted^b^ OR (95% CI)	1.00	1.06 (0.67–1.67)	1.31 (0.82–2.10)	0.25
				
Triglycerides ≥150 mg/dl				
No. of cases/subjects	45/168	36/193	40/193	
Adjusted^a^ OR (95% CI)	1.00	0.64 (0.38–1.05)	0.61 (0.37–1.02)	0.06
Adjusted^b^ OR (95% CI)	1.00	0.60 (0.35–1.02)	0.53 (0.31–0.90)	0.02

High-density lipoprotein cholesterol <40 mg/dl in men and <50 mg/dl in women				
No. of cases/subjects	27/168	20/193	27/193	
Adjusted^a^ OR (95% CI)	1.00	0.62 (0.33–1.16)	0.92 (0.50–1.68)	0.77
Adjusted^b^ OR (95% CI)	1.00	0.56 (0.29–1.07)	0.69 (0.37–1.31)	0.27

Systolic blood pressure ≥130 mm Hg or diastolic blood pressure ≥85 mm Hg or hypertension treatment				
No. of cases/subjects	62/168	61/193	49/193	
Adjusted^a^ OR (95% CI)	1.00	0.97 (0.61–1.55)	0.79 (0.48–1.28)	0.34
Adjusted^b^ OR (95% CI)	1.00	0.89 (0.55–1.45)	0.82 (0.49–1.36)	0.44

Fasting plasma glucose ≥100 mg/dl or diabetes treatment				
No. of cases/subjects	77/168	68/193	79/193	
Adjusted^a^ OR (95% CI)	1.00	0.74 (0.48–1.16)	0.86 (0.55–1.35)	0.53
Adjusted^b^ OR (95% CI)	1.00	0.74 (0.47–1.18)	0.81 (0.51–1.30)	0.40

^a^Adjusted for age and sex.

^b^Adjusted for age, sex, total energy intake, physical activity, and smoking and drinking habits.

Table 3 shows sex- and age-adjusted and multivariate-adjusted associations of coffee consumption with the prevalence of MetS and its components, as defined using JASSO criteria. Using these criteria, the prevalence of MetS was 77/554 (13.9%). In multivariate-adjusted models, moderate coffee consumption (1.5 to <3 cups/day) was significantly associated with lower prevalence of MetS (OR = 0.52, 95% CI 0.27–0.97). Regarding each component of MetS, participants who drank more coffee (≥3 cups/day) had a significantly lower OR for dyslipidemia (OR = 0.54, 95% CI 0.33–0.89). Moderate coffee consumption (1.5 to <3 cups/day) was associated with a lower OR for high plasma glucose (OR = 0.51, 95% CI 0.28–0.93). The *P* for trend for the association with coffee consumption was 0.09 for MetS and 0.02 for dyslipidemia.

**Table 3.** Associations of coffee consumption with the prevalence of metabolic syndrome and its components according to JASSO criteria (No. = 554)

	Coffee consumption (cups/day)			*P* for trend

	<1.5	≥1.5 and <3	≥3	
Metabolic syndrome				
No. of cases/subjects	32/168	21/193	24/193	
Adjusted^a^ OR (95% CI)	1.00	0.58 (0.31–1.05)	0.62 (0.34–1.12)	0.10
Adjusted^b^ OR (95% CI)	1.00	0.52 (0.27–0.97)	0.59 (0.32–1.10)	0.09

Waist circumference ≥85 cm in men and ≥90 cm in women				
No. of cases/subjects	76/168	72/193	91/193	
Adjusted^a^ OR (95% CI)	1.00	0.81 (0.52–1.29)	0.94 (0.60–1.48)	0.83
Adjusted^b^ OR (95% CI)	1.00	0.77 (0.48–1.23)	0.93 (0.58–1.49)	0.81

Triglycerides ≥150 mg/dl or high-density lipoprotein cholesterol <40 mg/dl				
No. of cases/subjects	54/168	44/193	49/193	
Adjusted^a^ OR (95% CI)	1.00	0.65 (0.40–1.04)	0.63 (0.39–1.01)	0.05
Adjusted^b^ OR (95% CI)	1.00	0.61 (0.37–1.00)	0.54 (0.33–0.89)	0.02

High-density lipoprotein cholesterol <40 mg/dl				
No. of cases/subjects	18/168	18/193	24/193	
Adjusted^a^ OR (95% CI)	1.00	0.93 (0.46–1.88)	1.09 (0.56–2.15)	0.79
Adjusted^b^ OR (95% CI)	1.00	0.83 (0.40–1.74)	0.84 (0.41–1.72)	0.65

Systolic blood pressure ≥130 mm Hg or diastolic blood pressure ≥85 mm Hg or hypertension treatment				
No. of cases/subjects	62/168	61/193	49/193	
Adjusted^a^ OR (95% CI)	1.00	0.97 (0.61–1.55)	0.79 (0.48–1.28)	0.34
Adjusted^b^ OR (95% CI)	1.00	0.89 (0.55–1.45)	0.82 (0.49–1.36)	0.44

Fasting plasma glucose ≥110 mg/dl or diabetes treatment				
No. of cases/subjects	40/168	22/193	27/193	
Adjusted^a^ OR (95% CI)	1.00	0.49 (0.27–0.87)	0.64 (0.36–1.13)	0.10
Adjusted^b^ OR (95% CI)	1.00	0.51 (0.28–0.93)	0.65 (0.36–1.17)	0.13

^a^Adjusted for age and sex.

^b^Adjusted for age, sex, total energy intake, physical activity, and smoking and drinking habits.

The results of Tables 2 and 3 did not change when percent energy intake from fat and frequency of green tea consumption were considered as potential confounders and were additionally adjusted for (data not shown).

Tables 4 and 5 show the associations between green tea consumption and the prevalence of MetS based on the NCEP ATP III and JASSO criteria, respectively. A higher green tea intake (≥4 cups/day) was not significantly associated with MetS or any of its components, regardless of whether the NCEP ATP III or JASSO criteria were used for diagnosis.

**Table 4.** Associations of green tea consumption with the prevalence of metabolic syndrome and its components according NCEP ATP III criteria (No. = 554)

	Green tea consumption (cups/day)			*P* for trend

	<1	≥1 and ≤3	≥4	
Metabolic syndrome				
No. of cases/subjects	16/89	60/310	38/155	
Adjusted^a^ OR (95% CI)	1.00	1.00 (0.55–1.90)	1.25 (0.64–2.50)	0.43
Adjusted^b^ OR (95% CI)	1.00	1.01 (0.54–1.95)	1.14 (0.57–2.34)	0.66

Waist circumference ≥90 cm in men and ≥80 cm in women				
No. of cases/subjects	30/89	115/310	62/155	
Adjusted^a^ OR (95% CI)	1.00	1.15 (0.70–1.93)	0.99 (0.56–1.77)	0.85
Adjusted^b^ OR (95% CI)	1.00	1.21 (0.72–2.07)	1.01 (0.56–1.83)	0.88

Triglycerides ≥150 mg/dl				
No. of cases/subjects	17/89	73/310	31/155	
Adjusted^a^ OR (95% CI)	1.00	1.31 (0.74–2.44)	1.28 (0.65–2.56)	0.55
Adjusted^b^ OR (95% CI)	1.00	1.38 (0.76–2.63)	1.18 (0.59–2.44)	0.76

High-density lipoprotein cholesterol <40 mg/dl in men and <50 mg/dl in women				
No. of cases/subjects	9/89	40/310	25/155	
Adjusted^a^ OR (95% CI)	1.00	1.28 (0.62–2.92)	1.61 (0.72–3.89)	0.24
Adjusted^b^ OR (95% CI)	1.00	1.23 (0.58–2.88)	1.49 (0.64–3.69)	0.35

Systolic blood pressure ≥130 mm Hg or diastolic blood pressure ≥85 mm Hg or hypertension treatment				
No. of cases/subjects	23/89	90/310	59/155	
Adjusted^a^ OR (95% CI)	1.00	0.97 (0.55–1.72)	1.28 (0.69–2.40)	0.33
Adjusted^b^ OR (95% CI)	1.00	1.01 (0.57–1.82)	1.21 (0.64–2.32)	0.49

Fasting plasma glucose ≥100 mg/dl or diabetes treatment				
No. of cases/subjects	41/89	123/310	60/155	
Adjusted^a^ OR (95% CI)	1.00	0.64 (0.39–1.05)	0.68 (0.38–1.20)	0.26
Adjusted^b^ OR (95% CI)	1.00	0.63 (0.38–1.07)	0.73 (0.40–1.32)	0.40

^a^Adjusted for age and sex.

^b^Adjusted for age, sex, total energy intake, physical activity, smoking and drinking habits, and coffee consumption.

**Table 5.** Associations of green tea consumption with the prevalence of metabolic syndrome and its components according to JASSO criteria (No. = 554)

	Green tea consumption (cups/day)			*P* for trend

	<1	≥1 and ≤3	≥4	
Metabolic syndrome				
No. of cases/subjects	11/89	41/310	25/155	
Adjusted^a^ OR (95% CI)	1.00	0.98 (0.49–2.09)	1.38 (0.64–3.14)	0.33
Adjusted^b^ OR (95% CI)	1.00	0.99 (0.49–2.17)	1.18 (0.53–2.75)	0.63

Waist circumference ≥85 cm in men and ≥90 cm in women				
No. of cases/subjects	37/89	145/310	57/155	
Adjusted^a^ OR (95% CI)	1.00	1.13 (0.68–1.88)	1.00 (0.56–1.81)	0.93
Adjusted^b^ OR (95% CI)	1.00	1.13 (0.67–1.90)	0.87 (0.48–1.60)	0.57

Triglycerides ≥150 mg/dl or high-density lipoprotein cholesterol <40 mg/dl				
No. of cases/subjects	20/89	90/310	37/155	
Adjusted^a^ OR (95% CI)	1.00	1.39 (0.80–2.48)	1.29 (0.68–2.48)	0.54
Adjusted^b^ OR (95% CI)	1.00	1.43 (0.81–2.61)	1.18 (0.61–2.33)	0.77

High-density lipoprotein cholesterol <40 mg/dl				
No. of cases/subjects	7/89	36/310	17/155	
Adjusted^a^ OR (95% CI)	1.00	1.49 (0.67–3.78)	1.74 (0.70–4.76)	0.27
Adjusted^b^ OR (95% CI)	1.00	1.44 (0.63–3.75)	1.64 (0.63–4.65)	0.34

Systolic blood pressure ≥130 mm Hg or diastolic blood pressure ≥85 mm Hg or hypertension treatment				
No. of cases/subjects	23/89	90/310	59/155	
Adjusted^a^ OR (95% CI)	1.00	0.97 (0.55–1.72)	1.28 (0.69–2.40)	0.33
Adjusted^b^ OR (95% CI)	1.00	1.01 (0.57–1.82)	1.21 (0.64–2.32)	0.49

Fasting plasma glucose ≥110 mg/dl or diabetes treatment				
No. of cases/subjects	14/89	49/310	26/155	
Adjusted^a^ OR (95% CI)	1.00	0.83 (0.43–1.68)	0.84 (0.40–1.81)	0.70
Adjusted^b^ OR (95% CI)	1.00	0.84 (0.43–1.71)	0.84 (0.39–1.86)	0.70

^a^Adjusted for age and sex.

^b^Adjusted for age, sex, total energy intake, physical activity, smoking and drinking habits, and coffee consumption.

## DISCUSSION

In the present study, coffee consumption was inversely correlated with the prevalence of MetS, as defined by the NCEP ATP III criteria, after adjustment for sex, age, total energy intake, leisure-time physical activity, and smoking and drinking habits. Earlier studies are inconsistent regarding the association of coffee consumption and MetS. Hino et al^15^ reported a significant inverse correlation in a cross-sectional study of 1902 Japanese older than 40 years. They also observed significant inverse correlations in all 5 MetS components (Japanese criteria) except low HDL cholesterol. During the preparation of this manuscript, Matsuura et al^16^ reported that habitual coffee consumption (≥4 cups/day) was associated with a significantly lower prevalence of MetS (Japanese criteria), and significantly lower blood pressure and serum triglycerides, in men. In contrast, no significant association was observed between coffee consumption and the incidence rate of MetS in a 9-year prospective study of 9514 subjects in the United States.^8^ In addition, coffee consumption was not significantly associated with MetS or any of its components in 283 Dutch men and women followed for 15 years.^17^ However, the age of that study population was rather young (27 years at baseline), and the prevalence of MetS at the completion of follow-up was only 8.1% among men and 0% among women.^17^

In our study, serum triglyceride level was significantly inversely correlated with coffee consumption. At least 4 cross-sectional studies performed in Japan, including 2 on MetS,^15^^,^^16^ reported an inverse correlation between coffee consumption and serum triglyceride level. In 4587 self-defense officials, consumption of instant but not brewed coffee was significantly correlated with lower serum triglyceride levels.^25^ There was also an inverse relationship between coffee drinking and serum triglyceride level in 1591 male office workers in Osaka.^26^ However, among 623 adults in Germany, there was a significant positive relation between serum caffeine, an important chemical component of coffee, and serum triglyceride level.^27^ It is possible that substances in coffee other than caffeine have biological effects that decrease serum triglyceride level. In randomized controlled trials conducted in the 1990s, administration of 5 to 6 cups/day of boiled coffee^28^ or lipids extracted from Arabica coffee beans^29^ significantly increased plasma or serum levels of total cholesterol and triglycerides. However, this lipid fraction is believed to be absent from filtered and instant coffee, which are the most common types of coffee in Japan.

Regarding low HDL cholesterol levels, the results of 2 clinical trials in the United States showed a significant increase in serum HDL cholesterol after consumption of 4 to 8 cups/day of filtered coffee for 8 weeks.^30^^,^^31^ However, in studies of Japanese populations that were conducted in the 1990s, no significant correlation was observed between coffee consumption and serum HDL cholesterol level.^25^^,^^26^ In the study by Hino et al,^15^ low HDL cholesterol was the only component of MetS that was not correlated with coffee consumption. Moreover, there was no significant correlation of intakes of caffeinated and decaffeinated coffee with plasma HDL cholesterol level in a sample selected from the 2 US cohort studies.^32^ In the present study population, the ORs for low HDL cholesterol levels were lower than unity (0.56 for 1.5 to <3 cups/day and 0.69 for >3 cups/day), but not significantly so.

There have been a large number of epidemiologic studies of the link between coffee consumption and glucose metabolism, and recent meta-analyses of cohort studies concluded that coffee and decaffeinated coffee may be protective against type 2 diabetes/impaired glucose tolerance and that habitual consumption of 1 cup/day of coffee was associated with an approximately 7% reduction in the risk of type 2 diabetes.^13^^,^^14^ The hypothesized biological mechanisms include reduced intestinal glucose absorption through inhibition of glucose-6-phosphate translocase 1, decreased hepatic output of glucose and the antioxidant effects of chlorogenic acid, and improved insulin sensitivity due to magnesium.^33^ In the present study, a significant inverse association with moderate coffee consumption (1.5 to <3 cups/day) was seen when a plasma glucose level of 110 mg/dl, but not 100 mg/dl, was used as the cut-off point. This may be due to the higher specificity of detecting impaired glucose tolerance/diabetes when 110 mg/dl is used as a cut-off point.

A cross-sectional study on self-defense officials in Japan showed a significant inverse correlation between coffee consumption and systolic and diastolic blood pressures.^34^ In addition, a Japanese cross-over study reported that a coffee intake of 3.3 to 3.6 cups/day for 4 weeks was associated with significant reductions in systolic and diastolic blood pressures in male habitual alcohol drinkers.^35^ A small but significant reduction in the risk of hypertension (7%–12%) was also observed among women who consumed 4 to 5 cups/day or at least 6 cups/day of caffeinated coffee in a large-scale cohort study in the United States.^36^ On the other hand, in a recent meta-analysis of 6 prospective studies in the United States and Europe, there was an inverse J-relation between coffee consumption and hypertension risk, and the pooled rate ratios of hypertension associated with a coffee intake of 3 to 5 cups/day and more than 5 cups/day were 1.07 (95% CI 0.96–1.20) and 1.08 (0.96–1.21), respectively, when an intake less than 1 cup/day was used as the reference.^37^ In another meta-analysis, caffeine administration resulted in an acute increase in systolic and diastolic blood pressures, but there was no prolonged increase or decrease in blood pressure in patients with hypertension, after coffee consumption of at least 3 cups/day for 2 weeks.^38^ In the present study, although high coffee consumption (≥3 cups/day) was correlated with significantly lower systolic and diastolic blood pressures when analyzed as a continuous variable, it was not correlated with hypertension prevalence, perhaps because inverse correlations were observed even within the normal range of blood pressures, and because loss of information occurred during the conversion from continuous to categorical variables.

Two cross-sectional data sets, 1 from Japanese adults^15^ and 1 from American Indians,^39^ showed inverse correlations between the frequency of coffee consumption and waist circumference. In a prospective study of US women, changes in the intakes of caffeinated and decaffeinated coffee were significantly associated with weight gain.^40^ An experimental study also showed that administration of coffee polyphenols increased energy expenditure and suppressed visceral fat accumulation in mice.^41^ In contrast, there was no relationship between frequency of coffee consumption and waist circumference among participants of the 2003–2004 National Health and Nutrition Examination Survey in the United States^42^ or the Amsterdam Growth and Health Longitudinal Study in the Netherlands.^17^ In our analysis, no significant correlation was found between coffee consumption and either waist circumference or body mass index. Differences in age, ethnicity, amount and type of coffee consumed, and use of additives such as sugar and milk, might account for the inconsistency. However, the precise reasons remain unclear.

In the current study, a significant inverse dose-response relationship between coffee consumption and MetS prevalence was observed when NCEP ATP III but not JASSO criteria were used for diagnosis. The reason may be that an essential JASSO criterion is increased waist circumference, which was not associated with coffee consumption.

Observational studies of general populations and intervention studies of obese adults showed beneficial effects of high intake of green tea, green tea extracts, and especially (−)-epigallocatechin-3-gallate, on weight reduction^18^^,^^43^ and other cardiovascular risk factors such as serum triglycerides^19^ and hypertension.^20^^,^^44^ In addition, a significant association between high green tea consumption (>6 cups/day) and reduced risk of diabetes was reported in a Japanese population.^21^ In the present study, however, there was no correlation between green tea consumption and the prevalence of MetS or any of its components. One explanation for the lack of a correlation in our study may be that the interindividual variation in green tea consumption was rather low, as indicated by the narrow interquartile ranges. In addition, the question on Japanese green tea included coarse tea, which contains relatively little (−)-epigallocatechin-3-gallate.

The current study has several limitations. First, because of the cross-sectional study design, the temporal relationship between coffee consumption and MetS development is obscure. It is also possible that the presence of chronic diseases led to modifications in dietary habits. For instance, persons who had received a diagnosis of diabetes might have refrained from drinking coffee with sugar. When participants receiving treatment for diabetes were excluded, the ORs for MetS (NCEP ATP III definition) associated with coffee consumption (1.5 to <3 cups/day and ≥3 cups/day) were 0.66 (95% CI 0.37–1.16) and 0.60 (95% CI 0.33–1.09), respectively (*P* for trend = 0.09, *n* = 516). Second, information on additives such as milk, sugar, and cream was lacking, and we could not account for these factors in the analysis. Third, we could not distinguish between intake of caffeinated and decaffeinated coffee, although use of decaffeinated coffee is not common in Japan. Fourth, information on coffee and green tea consumption was based on self-reporting, and some degree of misclassification is inevitable. Finally, the number of study participants was rather small.

In summary, these cross-sectional data showed an inverse correlation between coffee consumption and MetS when NCEP ATP III criteria were used for diagnosis, mainly due to the lower prevalence of hypertriglyceridemia. This association warrants further prospective studies for the purpose of causal inference.

## ONLINE ONLY MATERIALS

The Japanese-language abstract for articles can be accessed by clicking on the tab labeled Supplementary materials at the journal website http://dx.doi.org/10.2188/jea.JE20120053.

Abstract in Japanese.
