# Supplementary material for: Inverse Correlation Between Coffee Consumption and Prevalence of Metabolic Syndrome: Baseline Survey of the Japan Multi-Institutional Collaborative Cohort (J-MICC) Study in Tokushima, Japan
Source: J Epidemiol. 2013 Jan 5;23(1):12–20. doi: 10.2188/jea.JE20120053 (PMC3700235; doi:10.2188/jea.JE20120053)
Supplement: Abstract in Japanese. [file je-23-012-s001.pdf]

コーヒー摂取とメタボリックシンドローム有病率との負の関連：日本多施設コーホート研究（J-MICC Study）徳島地区ベースライン調査

高見栄喜[1]、中本真理子[1]、上村浩一[1]、勝浦桜子[1]、山口美輪[1]、日吉峰麗[1]、澤近房和[1]、十田朋也[1]、有澤孝吉[1]

[1]徳島大学大学院ヘルスバイオサイエンス研究部予防医学分野

【背景】コーヒーおよび緑茶の摂取量とメタボリックシンドローム（MetS)との間に関連があるかどうかは明らかになっていない。

【方法】この断面調査では、日本多施設共同コーホート研究（J-MICC Study)徳島地区ベースライン調査に参加した 554 名を対象とした。コーヒーと緑茶の摂取量は、質問票で測定した。MetS の診断には、National Cholesterol Education Program Adult Treatment Panel Ⅲ（NCEP ATPⅢ)および日本肥満学会（JASSO)の基準を用いた。コーヒーおよび緑茶の摂取量と MetS およびその構成因子の有病率との間の関連を調べるためにロジスティック回帰分析を用いた。

【結果】性、年齢、その他の交絡因子を調整した後、コーヒー摂取量と NCEP ATPⅢの MetS 基準により診断した MetS の有病率との間で有意な負の関連を認めた（P for trend= 0.03)。またコーヒーの高摂取群で中性脂肪高値のオッズ比（OR)の有意な低下を認めたが (P for trend= 0.02)、腹囲の増加や血圧の上昇との関連は認めなかった。JASSO の基準を用いた場合は、コーヒーの中摂取群（1.5 杯/日以上 3 杯/日未満)で高血糖のオッズ比の有意な低下を認めた（OR= 0.51、95%信頼区間: 0.28-0.93)。緑茶の摂取量と MetS およびその構成因子との間には有意な関連を認めなかった。

【結論】コーヒー摂取が、主として血清中性脂肪濃度との負の相関により、NCEP ATPⅢ基準による MetS の有病率の低下と関連していることが示唆された。今後、因果関係の検証のため、前向き研究などのさらなる研究が必要である。

キーワード：メタボリックシンドローム、コーヒー、中性脂肪値、血糖値、緑茶
